# Enabling new frontiers of nanophotonics with metamaterials, photonic crystals, and plasmonics

**DOI:** 10.1515/nanoph-2024-0100

**Published:** 2024-03-15

**Authors:** Trevon Badloe, Junsuk Rho

**Affiliations:** Graduate School of Artificial Intelligence, Pohang University of Science and Technology (POSTECH), Pohang 37673, Republic of Korea; Department of Mechanical Engineering, Pohang University of Science and Technology (POSTECH), Pohang 37673, Republic of Korea; Department of Chemical Engineering, Pohang University of Science and Technology (POSTECH), Pohang 37673, Republic of Korea; Department of Electrical Engineering, Pohang University of Science and Technology (POSTECH), Pohang 37673, Republic of Korea; POSCO-POSTECH-RIST Convergence Research Center for Flat Optics and Metaphotonics, Pohang 37673, Republic of Korea

Nanophotonics is a cutting-edge interdisciplinary field that merges state-of-the-art nanotechnology with the traditional field of optics. Light–matter interactions at the nanoscale differ greatly from the behaviors we see at the macroscale in our daily lives, and the field of nanophotonics aims to develop new ways to generate, manipulate, and detect light by uncovering new physics and pioneering innovative engineering solutions. Recent advancements in nanofabrication, machine learning, and the understanding of fundamental physics have pushed the boundaries of nanophotonics with applications in communications, sensing, imaging, and beyond.

*The International Conference on Metamaterials, Photonics Crystals, and Plasmonics* (META) is an annual conference that brings together researchers from across the globe to share the latest developments and pioneering research in the field. A broad range of topics are covered, from topological photonics, sustainable materials, quantum photonics, biophotonics, and machine learning, to name just a few. The latest conference, META’23, was held over 4 days in Paris, France (July 18–21, 2023), with options for remote viewing of oral presentations, as well as hybrid online and offline poster sessions. This special issue “*Enabling* *New Frontiers of Nanophotonics through Metamaterials, Photonic Crystals, and Plasmonics*” introduces a selection research and review articles from the conference.

At the most fundamental level, the creation of novel nanophotonic devices requires the convergence of innovative nanofabrication techniques with novel design strategies. The growth of computational power available to researchers has opened up new avenues of design to explore, in particular the concept of inverse design. Furthermore, as the boundaries of 3D metasurfaces are being pushed to their limits, the next obvious step is to add an extra dimension to create 3D shapes. Proximity-field nanopatterning (PnP) is a lithography technique that enables the nanofabrication of such 3D shapes, and therefore, corresponding design methodologies must be investigated and developed. With that in mind, Chang et al. [1] review the latest advancements in the inverse design used in PnP, including the adjoint method, particle swarm optimisation, and machine learning to create interference patterns with desired to produce the desired optical properties. Next generation communications could enable new applications for industry and the heath sector, as well as for the general public. Integration of photonics at terahertz frequencies has the potential to disrupt current technology in terms of both data transmission speeds and innovative measurement techniques. In [2], Kang et al. review the latest developments in frequency comb measurements for sixth generation (6G) terahertz nano- and micro-photonics with metamaterials. They discuss the critical requirements for 6G devices and provide an outlook on the emerging trends for next generation communications, including comparisons between microwave and nanophotonic devices for high-resolution THz sensors, with a keen focus on the latest progress of frequency comb measurements in the THz regime. They suggest that the integration of nanophotonic devices could hold the potential to revolutionise high-density 6G wireless communications in various applications, such as satellites, drones, and Internet of Things devices.

On the topic of 6G communications and the transportation of information through electromagnetic (EM) waves, groundbreaking work on signal propagation through surface waves from Smolyaninov et al. is presented [3]. They develop of theory of EM waves propagating in media with gradients of lossy dielectric permittivity and magnetic permeability to prove unique low loss propagating surface wave solutions. Furthermore, they demonstrate several analytical examples of geometries that could provide practical applications of their theoretical investigations, from long radio wave communications underwater, to ultrashort wavelength UV nanophotonics. Waveguides are a key component for optical communications, and in [4], Akhter et al. demonstrate all-optical spatial mode-cleaning in non-Hermitian waveguides. By modulating the refractive index and gain/loss along graded index multimodal waveguides, they show that almost monomodal propagation can be achieved. They prove their work numerically in 1D waveguides and propose a generalised method for 2D waveguides to improve beam spatial quality. Optical fibers are a well-known way of transmitting information at the speed of light by exploiting the concept of total internal reflection. As losses in optical fibers are extremely detrimental to performance, Zhang et al. [5] demonstrate how an epsilon near zero (ENZ) material such as indium tin oxide can be employed as the cladding in an air-core fiber to reduce propagation losses. They uncover that these losses can be further reduced through the use of ENZ materials with lower losses.

Photoluminescence (PL) is another topic where nanophotonics has a strong influence such as for manipulating emission and decay rates in novel ways. In [6], Choi et al. achieve an increased Purcell effect using organic monolithic molecular aggregate films. They exploit ENZ and epsilon-near-pole regions to increase and decrease the photonic density of states. Additionally, Chen et al. [7] investigate the PL emission and Raman enhancement between two closely spaced gold nanospheres using tip-enhanced Raman spectroscopy (TERS), analytically and experimentally. They uncover a rapid increase in Raman and PL signals in gaps smaller than 10 nm. Their analytic model neglects non-classical effects, but correlates well with the experimental results, proving its ability to capture the important interactions in the processes. The detection of photons is another research direction that is greatly influenced by nanophotonics, to uncover novel ways to sense and detect light as efficiently as possible. Accordingly, Kim et al. [8] develop a strategy to exploit hot carriers in Au/Si Schottky junctions for photodetection at NIR wavelengths. They employ extremely thin layers of Au to achieve hot carrier generation and injections into the Si, with thicker layers of Au acting as electrodes to experimentally demonstrate the application of a commercial beam profiler.

Acoustics is a research field founded on the basis of mechanical waves, such as sound waves, rather than EM waves. Despite this important difference, many similarities exist between the two disciplines. Park et al. [9] combine the two domains to demonstrate ultrafast acousto-optic modulation through the conversion of light and sound in van der Waals thin films of Bi_2_Se_3_. Ultrafast lasers induce interlayer vibrations in the GHz regime, for narrow bandwidth applications. Their findings hold promise for high performance signal detection and processing in the NIR spectral range. 2D materials offer an exciting base for nanophotonic research due to their extremely interesting optical and electrical properties. In [10], Kim et al. study the characteristics of the interface between two lateral transition metal dichalcogenide heterostructures, namely between MoS_2_ and WS_2_. They observe notable variations in the composition of the nanoscale alloy through TERS to uncover the disordered interface at the heterostructure. An interesting branch of nanophotonics is topological physics, a field which stemmed from a branch of mathematics, but contributes significantly in many nanophotonic processes and systems. Topological insulators are one of the most well-known examples, with the exotic property of not allowing electricity to conduct in their bulk, while electrons are free to move along their edges and surfaces. Park et al. [11] investigated the topological phase transition of nodal lines and surface states in photonic crystals. They show that the deformation of photonic crystals causes topological phase transitions in the nodal lines, and that the Euler class can be used to theoretically predict their stability.

Metasurfaces, the quasi-2D counterparts of metamaterials, continue to be a hot topic of interest due to their unique capability of manipulating light at the nanoscale to produce optical properties that cannot be found in nature. Plasmonic metasurfaces that exploit the collective oscillation of electrons at metal-dielectric interfaces continue to produce intriguing new research. Lee et al. [12] expand Bethe’s theory of the scattering produced by a small void in a thin perfect electric conductor to ultraviolet (UV) wavelengths. Interestingly, they realise that as the conductivity of metalens diminishes in the UV range, the interband transition of silicon earmarks it as a candidate material for UV plasmonics. Additionally, due to the high-Q and sensitive nature of plasmonic resonances, they provide an ideal system for sensing applications; however this limits their bandwidth significantly. Huang and Wu [13] provide a refractive index sensing platform by measuring the far-field intensity ratio between the energy in the beam deflected by the plasmonic gradient metasurfaces and the specular reflection. Such sensors hold great potential for highly sensitive measurements in biomedical diagnostics and environmental monitoring.

One of the key drawbacks of metasurfaces is that after they are designed and fabricated, their optical properties are fixed. However, through the implementation of novel materials, metasurfaces with variable optical properties can be created. One such material is the phase change material VO_2_ that Liao et al. [14] utilise for switchable metaholography for optical encryption. They design meta-atoms to provide different phase modulations depending on the state of the VO_2_, allowing for thermal switching between two distinct holographic images based on the temperature of the metasurface. One of the most promising methods of modulating metasurfaces is through the use of conducting polymers that have electrically tunable optical properties. Polyaniline (PANI) is one of such polymers that Ko et al. [15] combine with plasmonics to create an electrochromic material at visible wavelengths. By coating Au/Si nanowires with PANI, they construct nanopixels that can be tuned from red, to green, and blue colours and prove an 8 bit encryption technique within a sub-1 V voltage. Another exciting research field is in nonlinear metasurfaces that offer a pathway for exploring nonlinear phenomena in planar geometries. This can further enhance the multiplexing potential of metasurfaces, but research is still required toward innovative new methods of creating nonlinear effects. Consequently, in [16], Yu et al. uncover giant nonlinear responses over a broadband range using semiconductors and nanocavities. They experimentally realise broadband second harmonic generation through the application of a bias voltage, opening up a new route for electrically tunable nonlinearities in metasurfaces.

To facilitate the experimentation of nanophotonics, nanofabrication techniques are of paramount importance. Advancements in nanofabrication and development of state-of-the-art methods to physically realise nanophotonic devices drives the process of science from computational calculations to real-life examples. The fabrication of metal-wire-embedded ZnO nanowires is uncovered by Kim et al. [17] through solution-processable metal wires. Without the need for vacuum conditions, nanoscale lithography, or etching, the process can be performed on any substrate, opening up the possibility of flexible polymer substates and large-scale production. Hollow nanoparticles have extremely interesting optical properties, but their fabrication is far from straightforward. In [18], Sánchez-Pérez et al. demonstrate the formation of hollow nanoparticles made of silver through the irradiation of ultrashort laser pulses for the first time. Their results support their earlier models for predicting the formation of hollow silver nanoparticles and identify the key role that silica plays in the expansion and stabilisation of the nanocavity. Such techniques could be potentially utilised for cavities in nanoparticles with various other metals such as gold or platinum, as well as ceramic hosts. Meanwhile, Caligiuri et al. [19] proved a method of creating bilayers of nanoporous metal films and showed their potential use as a plasmonic metamaterial. The Au–Ag films show optical properties that significantly differ from the individual constituents. As their process can be generally applied to the deposition of any metals, their work opens the door for research into multilayers of combinations of nanoporous metals. Finally, work by Jeong et al. [20] reveals how they create a one-step stencil lithography technique to fabricate 3D surface lattice plasmon resonances in inclined nanostructures. The symmetry-breaking that the inclined structures provide allows for an increase of the polarizability of the created metasurfaces which allows for an increased directionality of the scattered field.

Last but not least, as machine learning and artificial intelligence (AI) spreads throughout society at an incredible pace with consumer products filled with AI that significantly enhances the user experience, the field of nanophotonics is also taking giant strides with regards to the implementation and convergence of optics with AI and machine learning. Various strategies have been proven for the inverse design of metamaterials and nanophotonic devices using AI, however there is still a large area of unexplored potential for the use of machine learning in the measurement of nanoscale measurements. Jiang et al. [21] therefore successfully develop a non-destructive approach of enabling ellipsometry measurements with neural networks to determine the profile of various nano-gratings, regardless of how they were fabricated. Another important avenue of research is to take advantage of the potential that photons of light have to perform massive linear operations in parallel to advance computational speed and reduce costs. Huang et al. [22] investigate a hybrid optical and digital neural network system to prove that the addition of an optical encoder produces a higher accuracy, at a high operation rate, and with an extremely low power requirement. This work could pave the way for the development of hybrid optical front-end and digital back-end systems with improved performance and efficiency.

We would finally like to express our warmest gratitude to all authors that have contributed to the groundbreaking research in this collection. We hope that both novice and experienced readers will be inspired by the contents of this special issue and will be motivated to continue the rapid progression of scientific discovery in the realm of nanophotonics.

## References

[1] Chang H., Kwon S., Bae G., Jeon S. (2024). Rational design of arbitrary topology in three-dimensional space via inverse calculation of phase modulation. *Nanophotonics*.

[2] Kang G. (2024). Frequency comb measurements for 6G terahertz nano/microphotonics and metamaterials. *Nanophotonics*.

[3] Smolyaninov I. I., Balzano Q., Smolyaninova V. N., Soloviova D. (2024). Electromagnetic signal propagation through lossy media via surface electromagnetic waves. *Nanophotonics*.

[4] Akhter M. N., Botey M., Herrero R., Staliunas K. (2024). Mode-cleaning in antisymmetrically modulated non-Hermitian waveguides. *Nanophotonics*.

[5] Zhang L., Love S., Anopchenko A., Lee H. W. H. (2024). Hollow core optical fiber enabled by epsilon-near-zero material. *Nanophotonics*.

[6] Choi K. R. (2024). Photoluminescence lifetime engineering via organic resonant films with molecular aggregates. *Nanophotonics*.

[7] Chen Y.-T., Liu Q., Schneider F., Brecht M., Meixner A. J., Zhang D. (2024). Photoluminescence emission and Raman enhancement in TERS: an experimental and analytic revisiting. *Nanophotonics*.

[8] Kim G. (2024). Scalable hot carrier–assisted silicon photodetector array based on ultrathin gold film. *Nanophotonics*.

[9] Park T. G. (2024). Ultrafast acousto-optic modulation at the near-infrared spectral range by interlayer vibrations. *Nanophotonics*.

[10] Kim D. H. (2024). Probing the multi-disordered nanoscale alloy at the interface of lateral heterostructure of MoS 2 –WS 2. *Nanophotonics*.

[11] Park H., Jones A., Kim M., Oh S. S. (2024). Topological phase transition and surface states in a non-Abelian charged nodal line photonic crystal. *Nanophotonics*.

[12] Lee D., Lee Y., Kim D.-S. (2024). Ultraviolet light scattering by a silicon Bethe hole. *Nanophotonics*.

[13] Huang S. H., Wu P. C. (2024). Exploring plasmonic gradient metasurfaces for enhanced optical sensing in the visible spectrum. *Nanophotonics*.

[14] Liao Y., Fan Y., Lei D. (2024). Thermally tunable binary-phase VO2 metasurfaces for switchable holography and digital encryption. *Nanophotonics*.

[15] Ko J. H. (2024). Electrochromic nanopixels with optical duality for optical encryption applications. *Nanophotonics*.

[16] Lee J., Park S., Hwang I., Boehm G., Belkin M. A. (2024). Broadband giant nonlinear response using electrically tunable polaritonic metasurfaces. *Nanophotonics*.

[17] Kim T. (2024). Mechanically processed, vacuum- and etch-free fabrication of metal-wire-embedded microtrenches interconnected by semiconductor nanowires for flexible bending-sensitive optoelectronic sensors. *Nanophotonics*.

[18] Sánchez-Pérez F. (2024). Formation of hollow silver nanoparticles under irradiation with ultrashort laser pulses. *Nanophotonics*.

[19] Caligiuri V. (2024). Dry synthesis of bi-layers nanoporous metal films as plasmonic metamaterial. *Nanophotonics*.

[20] Jeong T. I. (2024). Three-dimensional surface lattice plasmon resonance effect from plasmonic inclined nanostructures via one-step stencil lithography. *Nanophotonics*.

[21] Jiang Z., Gan Z., Liang C., Li W. (2024). Generic characterization method for nano-gratings using deep-neural-network-assisted ellipsometry. *Nanophotonics*.

[22] Huang L., Tanguy Q. A. A., Fröch J. E., Mukherjee S., Böhringer K. F., Majumdar A. (2024). Photonic advantage of optical encoders. *Nanophotonics*.

